# Mesmerism Forty Years Ago

**Published:** 1887-10

**Authors:** Harriet Martineau

**Affiliations:** Tynemouth


### MESMERISM FORTY YEARS AGO.

A half century ago, Harriet Martineau, the popular English Authoress, was deeply afflicted by a painful disease which defied the skill of the most eminent physicians, and rendered her an almost helpless invalid for years. After experimenting with all the usual means of cure, attainable at the hands of the more skilful medical practitioners of her time, without appreciable benefit ; as a *dernier resort*, Miss Martineau was induced to try the effect of mesmerism in her case. As a result she was completely restored to health, and rather from a sense of duty, than otherwise, Miss Martineau gave the public the benefit of her experiences in a series of seven letters originally published in the *London Athenæum*, and subsequently in pamphlet form by the well known New York house of Harper and Brothers in 1845. As very much that Miss Martineau says of mesmerism notably in the last of these letters, applies with equal force to some of the more recent discoveries of our times, we have concluded to republish the seventh letter in these columns.

### LETTER VII.

TYNEMOUTH, November 28, 1844.

"MANY persons suppose that when the truth, use and beauty of Mesmerism are established, all is settled ; that no further ground remains for a rejection of it. My own late experience, and my observation of what is passing abroad, convince me that this is a mistake. I know that there are many who admit the truth and function of Mesmerism, who yet discountenance it. I know that the repudiation of it is far more extensive than the denial. It gives me pain to hear this fact made the occasion of contemptuous remark, as it is too often by such as know Mesmerism to be true. The repudiation I speak of proceeds from minds of a high order ; and their superstition (if superstition it be) should be encountered with better weapons than the arrogant compassion which I have heard expressed.

I own I have less sympathy with those who throw down their facts before the world, and then despise all who will not be in haste to take them up, than with some I know of, who would seriously rather suffer to any extent, than have recourse to relief which they believe unauthorized ; who would rather that a mystery remain sacred than have it divulged for their own benefit : who tell me to my face that they would rather see me sent back to my couch of pain than witness any tampering with the hidden things of Providence. There is a sublime rectitude of sentiment here, which commands and wins one's reverence and sympathy ; and if the facts of the history and condition of Mesmerism would bear out the sentiment, no one would more cordially respond to it than I—no one would have been more scrupulous about procuring recovery by such means—no one would have recoiled with more fear and disgust from the work of making known what I have experienced and learned. But I am persuaded that a knowledge of existing facts clears up the duty of the case, so as to prove that the sentiment must, while preserving all its veneration and tenderness, take a new direction, for the honor of God and the safety of man.

Granting to all who wish, that the powers and practice of Mesmerism (for which a better name is sadly wanted) are as old as man and society ; that from age to age there have been endowments and functions sacred from popular use, and therefore, committed by providential authority to the hands of a sacred class ; that the existence of mysteries ever has been, and probably must ever be, essential to the spiritual welfare of man : that there should ever be a powerful sentiment of sanctity investing the subject of the ulterior powers of immortal beings in their mortal state ; that it is extremely awful to witness, and much more to elicit, hidden faculties, and to penetrate by their agency into regions of knowledge otherwise unattainable ;—admitting all these things, still the facts of the present condition of Mesmerism in this country, and on two continents, leave to those who know them, no doubt of the folly and sin of turning away from the study of the subject. It is no matter of choice whether the subject shall remain sacred—a deposit of mystery in the hands of the church—as it was in the Middle Ages, and as the Pope and many Protestants would have it still. The Pope has issued an edict against the study and practice of Mesmerism in his dominions ; and there are some members of the Church of England who would have the same suppression attempted by means of ecclesiastical and civil law at home. But for this it is too late: the knowledge and practice are all abroad in society ; and they are no more to be reclaimed than the waters, when out in floods, can be gathered back into reservoirs. The only effect of such prohibitions would be to deter from the study of Mesmerism, the very class who should assume its administration, and to drive disease, compassion and curiosity into holes and corners to practice as a sin what is now done openly and guiltlessly, however recklessly, through an ignorance for which the educated are responsible. The time is past for facts of natural philosophy to be held at discretion by priesthoods ; for any facts which concern all human beings to be a deposit in the hands of any social class. Instead of re-enacting the scenes of old—setting up temples with secret chambers, oracles, and miraculous ministrations—instead of reviving the factitious sin and cruel penalties of witchcraft, (all forms assumed by mesmeric powers and faculties in different times), instead of exhibiting false mysteries in an age of investigation, it is clearly our business to strip false mysteries of their falseness, in order to secure due reverence to the true, of which there will ever be no lack. Mystery can never fail while man is finite: his highest faculties of faith will, through all time and all eternity, find ample exercise in waiting on truths above his ken: there will ever be in advance of the human soul, a region "dark through excess of light;" while all labor spent on surrounding clear facts with artificial mystery is just so much profane effort spent in drawing minds away from the genuine objects of faith. And look at the consequences! Because philosophers will not study the facts of that mental *rapport* which takes place in Mesmerism, whereby the mind of the ignorant often gives out in echo the knowledge of the informed, we have claims of inspiration springing up right and left. Because medical men will not study the facts of the mesmeric trance, nor ascertain the extremest of its singularities, we have tales of Estaticas, and of sane men going into the Tyrol and elsewhere to contemplate, as a sign from heaven, what their physicians ought to be able to report of at home as natural phenomena easily producible in certain states of disease. Because physiologists and mental philosophers will not attend to facts from whose vastness they pusillanimously shrink, the infinitely delicate mechanism and organization of brain, nerves and mind are thrown as a toy into the hands of children and other ignorant persons, and of the base. What, again, can follow from this but the desecration, in the eyes of the many, of things which ought to command their reverence ? What becomes of really divine inspiration when the commonest people find they can elicit marvels of prevision and insight ? What becomes of the veneration for religious contemplation when Estaticas are found to be at the command of very unhallowed—wholly unauthorized hands ? What becomes of the respect in which the medical profession ought to be held, when the friends of the sick and suffering, with their feelings all alive, see the doctors' skill and science overborne and set aside by means at the command of an ignorant neighbor—means which are all ease and pleasantness ? How can the profession hold its dominion over minds, however backed by law and the opinion of the educated, when the vulgar see and know that limbs are removed without pain, in opposition to the will of the doctors, and in spite of their denial of the facts ? What avails the decision of a whole College of Surgeons that such a thing could not be, when a whole town full of people know that it was ? Which must succumb, the learned body or the fact ? Thus are objects of reverence desecrated, not sanctified, by attempted restriction of truth, or of research into it. Thus are human passions and human destinies committed to reckless hands, for sport or abuse. No wonder if somnambules are made into fortune-tellers—no wonder if they are made into prophets of fear, malice and revenge, by reflecting in their somnambulism the fear, malice and revenge of their questioners ; no wonder if they are made even ministers of death, by being led from sick bed to sick bed in the dim and dreary alleys of our towns, to declare which of the sick will recover, and which will die ! Does any one suppose that powers so popular, and now so diffused, can be interdicted by law—such oracles silenced by the reserve of the squeamish—such appeals to human passions hushed—in an age of universal communication, by the choice of a class or two, to be themselves, dumb ? No : this is not the way. It is terribly late to be setting about choosing a way, but something must be done; and that something is clearly for those whose studies and art relate to the human frame to take up, earnestly and avowedly, the investigation of this weighty matter; to take its practice into their own hands, in virtue of the irresistible claim of qualification. When they become the wisest and the most skilful in the administration of Mesmerism, others, even the most reckless vulgar, will no more think of interfering than they now do of using the lancet, or operating on the eye. Here, as elsewhere, knowledge is power. The greater knowledge will ever insure the superior power. At present, the knowledge of Mesmerism, superficial and scanty as it is, is out of the professional pale. When it is excelled by that which issues from within the professional pale, the remedial and authoritative power will reside where it ought; and not till then. These are the chief considerations which have caused me to put forth these letters in this place ;—an act which may seem rash to all who are unaware of the extent of the popular knowledge and practice of Mesmerism. . . .

As for the frequent objection brought against inquiry into Mesmerism, that there should be no countenance of an influence which gives human beings such power over one another, I really think a moment's reflection, and a very slight knowledge of Mesmerism would supply both the answers which the objection requires. First, it is too late, as I have said above ; the power is abroad, and ought to be guided and controlled. Next, this is but one addition to the powers we have over one another already ; and a far more slow and difficult one than many which are safely enough possessed. Every apothecary's shop is full of deadly drugs—every workshop is full of deadly weapons—wherever we go, there are plenty of people who could knock us down, rob, and murder us; wherever we live there are plenty of people who could defame and ruin us. Why do they not ? Because moral considerations deter them. Then bring the same moral considerations to bear on the subject of Mesmerism. If the fear is of laying victims prostrate in trance, and exercising spells over them, the answer is, that this is done with infinitely greater ease and certainty by drugs than it can ever be by Mesmerism; by drugs which are to be had in every street. And as sensible people do not let narcotic drugs lie about in their houses, within reach of the ignorant and michievous, so would they see that Mesmerism was not practiced without witnesses and proper superintendence. It is a mistake, too, to suppose that Mesmerism can be used at will to strike down victims, helpless and unconscious, as laudanum does, except in cases of excessive susceptibility from disease; cases which are, of course, under proper ward. The concurrence of two parties is needful in the first place, which is not the case in the administration of narcotics: and then the practice is very uncertain in its results on most single occasions; and again, in the majority of instances; it appears that the intellectual and moral powers are more, and not less vigorous than in the ordinary state. As far as I have any means of judging, the highest faculties are seen in their utmost perfection during the mesmeric sleep; the innocent are stronger in their rectitude than ever, rebuking levity, reproving falsehood and flattery, and indignantly refusing to tell secrets, or say or do anything they ought not; while the more faulty confess their sins, and grieve over and ask pardon for their offences. The volitions of the Mesmerist may actuate the movements of the patient's limbs, and suggest the material of his ideas; but they seem unable to touch his *morale*. In this state the *morale* appears supreme, as it is rarely found in the ordinary condition. If this view is mistaken, if it is founded on too small a collection of facts, let it be brought to the test and corrected. Let the truth be ascertained and established; for it cannot be extinguished, and it is too important to be neglected.

And now one word of respectful and sympathizing accost unto those reverent and humble spirits who painfully question men's right to exercise faculties whose scope is a new region of insight and foresight. They ask whether to use these faculties be not to encroach upon holy ground, to trespass on the precincts of the future and higher life. May I inquire of these in reply, what they conceive to be the divinely appointed boundary of our knowledge and our powers? Can they establish, or indicate, any other boundary than the limit of the knowledge and powers themselves? Has not the attempt to do so, failed from age to age? Is it not the most remarkable feature of the progress of time, that, in handing over the future into the past, he transmutes its material incessantly, and without pause, converting what truth was mysterious, fearful, impious to glance at, into that which is safe, beautiful and beneficent to contemplate and use,—a clearly consecrated gift from the Father of all to the children who seek the light of his countenance. Where is his pleasure to be ascertained but in the ascertainment of what he gives and permits, in the proof and verification of what powers he has bestowed on us, and what knowledge he has placed within our reach? While regarding with shame all pride of intellect, and with fear the presumption of ignorance, I deeply feel that the truest humility is evinced by those who most simply accept and use the talents placed in their hands; and that the most childlike dependence upon their Creator appears in those who fearlessly apply the knowledge he discloses to the furtherance of that great consecrated object, the welfare of the family of man.

HARRIET MARTINEAU.

